# Targeting Gene-Viro-Therapy with AFP driving Apoptin gene shows potent antitumor effect in hepatocarcinoma

**DOI:** 10.1186/1423-0127-19-20

**Published:** 2012-02-09

**Authors:** Kang-Jian Zhang, Jing Qian, Shi-Bing Wang, Yi Yang

**Affiliations:** 1 State key Laboratory of Molecular Cell Biology, Institute of Biochemistry and Cell Biology, Shanghai Institutes for Biological Sciences; The Chinese Academy of Sciences, Shanghai 200031, China; 2 Xinyuan Institute of Medicine and Biotechnology, College of Life Sciences, Zhejiang Sci-Tech University, Hangzhou, Zhejiang Province, China; 3 School of Life Sciences, Institutes of Biomedical Sciences, Fudan University, Shanghai, China

**Keywords:** AD55-Apoptin, apoptosis, antitumor effect, hepatocelluar carcinoma, Cancer targeting gene-viro-therapy

## Abstract

**Background:**

Gene therapy and viral therapy are used for cancer therapy for many years, but the results are less than satisfactory. Our aim was to construct a new recombinant adenovirus which is more efficient to kill hepatocarcinoma cells but more safe to normal cells.

**Methods:**

By using the Cancer Targeting Gene-Viro-Therapy strategy, Apoptin, a promising cancer therapeutic gene was inserted into the double-regulated oncolytic adenovirus AD55 in which E1A gene was driven by alpha fetoprotein promoter along with a 55 kDa deletion in E1B gene to form AD55-Apoptin. The anti-tumor effects and safety were examined by western blotting, virus yield assay, real time polymerase chain reaction, 3-(4,5-dimethylthiazol-2-yl)-2, 5-diphenyltetrazolium bromide assay, Hoechst33342 staining, Fluorescence-activated cell sorting, xenograft tumor model, Immunohistochemical assay, liver function analysis and Terminal deoxynucleotidyl transferase mediated dUTP Nick End Labeling assay.

**Results:**

The recombinant virus AD55-Apoptin has more significant antitumor effect for hepatocelluar carcinoma cell lines (in vitro) than that of AD55 and even ONYX-015 but no or little impair on normal cell lines. Furthermore, it also shows an obvious in vivo antitumor effect on the Huh-7 liver carcinoma xenograft in nude mice with bigger beginning tumor volume till about 425 mm3 but has no any damage on the function of liver. The induction of apoptosis is involved in AD55-Apoptin induced antitumor effects.

**Conclusion:**

The AD55-Apoptin can be a potential anti-hepatoma agent with remarkable antitumor efficacy as well as higher safety in cancer targeting gene-viro-therapy system.

## Background

Hepatocellular carcinoma (HCC) is the third most common cause of cancer death in china and the fifth most prevalent male cancer worldwide. Routine curative therapies such as liver transplantation and surgical resection are offered to only limited patients. Other treatments include chemotherapy, radiotherapy, thermotherapy and so on may be beneficial for unresectable HCC, but recurrence is frequent and the long term survival rate remains poor [1]. Therefore, new effective and efficient therapies are urgently needed.

Gene therapy exhibits a promising advantage for many diseases such as Leber's congenital amaurosis, X-linked adrenoleukodystrophy and"Bubble boy" disease, which was selected as the one of top ten news in the journal of "sicence"[2]. However, it is not efficient for cancer therapy by delivering a single therapeutic gene because of polygenes related disregulated pathways in cancer cells. Recently, "Cancer Targeting Gene-Viro-Therapy" is an attractive strategy for cancer gene therapy, the principle of which is to insert anti-tumor genes into oncolytic virus [3]. As oncolytic virus replicating in tumor cells, the therapeutic gene simultaneously gets amplification, ultimately exhibits an enhanced effect on killing tumor cells. Conditional replicative adenoviruse is mostly used in this strategy by virtue of its ability to transfer foreign genes efficiently [4] and replicates selectively in cancer cells and destroys them [5]. Basically two main strategies are used to make their replication cancer-specific. The first involves deletion of viral genes that are dispensable in tumor cells but not in normal cells, such as ONYX-015 or ZD55 [6] which deleted E1B 55 kDa gene which control the viral mRNA transport [7]. The second is the replacement of viral promoters with tumor or tissue-specific promoters. Paul Hallenbec [8] have pioneered the efforts in this direction by using α-fetoprotein (AFP) promoters to drive the adenovirus E1A gene to treat hepatocellular carcinoma. AFP is expressed abundantly in fetal liver cells but not in normal adult liver cells. However, AFP is frequently re-expressed in HCC and correlated with disease progression. Approximately over 70% of primary HCC has actived AFP protein [9]. Due to the specific expression spectrum of AFP, AFP promoter was extensively used as hepatocarcinoma targetting promoter to drive the adenovirus E1A gene [8,10] or directly drive suicide genes such as herpes simplex virus thymidine kinase (HSV-tk) [11,12].

Apoptin, a chicken anemia virus (CAV)-derived protein, can induce apoptosis in a large panel of human transformed and malignant cells but not in normal cells [13]. It shows to be independent of tumor-suppressor gene p53 [14] and cannot be inhibited by oncogene Bcr-abl as well as even sometimes stimulated by over expression of the apoptosis inhibitor Bcl-2 [15-17]. In short, Apoptin is a promising and ideal agent for cancer gene therapy owing to its intrinsic specificity and the inherent low toxicity, although the mechanism has not yet been fully elucidated. Several studies have already shown the excellent efficacy and safety of Apoptin in cancer gene therapy by various ways from using TAT, PTD4-Apoptin fusion protein [18,19] to recombinant parvoviruses, adenoviruses and poxviruses directly harbored *apoptin *gene [20-22].

Previous studies in our laboratory have shown exceptional merit of Cancer Targeting Gene-Viro-Therapy, compared to gene therapy or virotherapy alone [23-25], which uses the conditionally replicating oncolytic adenovirus (CRAd) to harbor the antitumor gene and gains tumor-specific therapeutic effect. In this study, we constructed a double regulated CRAd "AD55" with both E1A gene driven by eAFP promoter (including SV40 enhancer at the upstream of 275 bp alpha-fetoprotein promoter, abbreviated to "eAFP") and E1B 55KD deletion, then the Apoptin gene expression cassette under controlling of human CMV promoter was inserted into AD55, formed AD55-Apoptin. The *in vitro *and *in vivo *results showed that AD55-Apoptin shows a remarkable and specific anti-hepatoma effect, furthermore it even can strongly inhibit the growth of advanced xenograft tumors with larger volume which always thought can't be easily stopped.

## Materials and methods

### Cell lines and culture conditions

HEK293 (human embryonic kidney cell line containing the E1A region of Ad5) was obtained from Microbix Biosystem Inc (Toronto, Canada). The HCC cell lines(HepG2, PLC, Hep3B and Huh-7) were from American Type Culture Collection (ATCC, Rockville, MD, USA), the other live cancer cell lines (SMMC-7721, BEL-7404) and normal liver cell lines(L-02, QSG-7701 and WI38) were obtained from Shanghai cell collection (shanghai, china). All these cell lines were cultured in Dulbecco's modified Eagle's medium (DMEM) supplemented with 10% heatinactivated fetal bovine serum (FBS), 4 mM glutamine,50 U/ml penicillin and 50 mg/ml streptomycin. All the cell lines cultured condition were at 37°C in 5% CO_2_.

### Construction of the plasmids, generation and purification of adenovirus vectors

The short fragment of pAd△E1P [26]digested by *EcoR*I and *Xba*I was cloned into pZD55 [6] to form the plasmid pAd△E1P-ZD55. The eAFP promoter was amplified from the plasmid pDRIVE03-SV40enh/AFP(h) v04 (InvivoGen Corp, USA; Catalog# pdrive-sv40-hafp), then the PCR product was subcloned into pAd△E1P-ZD55 by *Xho*I and *SnaB *I to form the plasmid pAD55. After inserting the apoptin expression cassette from pCA13-apoptin (JieRui biotech Corp, shanghai, China) into it, the plasmid pAD55-Apoptin was obtained. The oncolytic viruses AD55-Apoptin and AD55-Apoptin were generated by homologous recombination of either pAD55-Apoptin or pAD55 and the adenovirus packaging plasmid pBHGE3 (Microbix Biosystems, Toronto, ON, Canada) in HEK293 cells. Each recombinant adenovirus was isolated through three rounds of plaque purification in HEK293 cells and purified by ultracentrifugation in a cesium chloride gradient. Moreover, Virus Titers were determined by TCID50 assay in HEK293 cells. Cells were infected with adenovirus at different doses at 37°C in humidified atmosphere containing 5% CO_2_.

### Cell viability assay

Cells were plated in 96-well plates and treated with various adenoviruses. After infection for four days with the indicated multiplicity of infection(MOI), cell survival rate was evaluated by a standard 3-(4,5-dimethylthiazol-2-yl)-2,5-diphenyltetrazolium bromide (MTT) assay(Sigma, St Louis, MO), Medium was removed and fresh medium containing 3-(4,5-dimethylthiazol-2-yl)-2,5-diphenyltetrazolium bromide (MTT, 5 mg/ml) was added to each well. The cells were incubated at 37°C for 4 h, after draw off the supernatant of each well carefully and then an equal volume 150 μl of DMSO was added to each well and mixed thoroughly on concentrating table for 10 minutes. The absorbance from the plates was read at 595 nm with a DNA Expert Microplate Reader Model GENios.

### Western blot analysis

To determine the expression of various proteins, Western blot analysis was performed as described standard protocol. Cells were harvested by trypsinization and resuspended in lysis buffer (62.5 mM Tris-HCl pH 6.8, 2% SDS, 10 mM glycerol, 1.55% dithiothreitol). The total protein concentration was determined by the BCA™Protein Assay kit (PIERCE) as described protocol. Then, protein samples were separated by 10-15% SDS-polyacrylamide gel electrophoresis and transferred to nitrocellulose membranes (Millipore). Membranes were blocked in 5% BSA solution and incubated with primary antibodies, then detected by the appropriate secondary fluorescent antibodies and checked out with an Odyssey Infrared imaging system (LI-COR Biosciences Inc, America). The primary antibodies used were anti-E1A (Abcam, Cambridge, UK), and anti-actin (Beyotime, Haimen, China).

### Real-time quantitative PCR to detect the relative mRNA level of Apoptin

Huh-7 cells or QSG-7701 cells were seeded in 6-well culture plates and then infected with AD55 or AD55-Apoptin at a MOI of 10 for 24, 48 and 96 hours, respectively. The mRNA was collected by using of Trizol at the indicated time and then reversed-Transcripted into cDNA (**ReverTra Ace**, Toyobo). Primers (Apoptin Forward primer: ACCATCAACGGTGTTCAGG; Apoptin Reverse primer: CAGCCACACAGCGATAGAG;GADPH Forward primer: CATCATCCCTGCCTCTACTG; GADPH Reverse primer: GCCTGCTTCACCACCTTC) were used to amplify apoptin or GAPDH gene according the standard procedures of real time PCR and obtained the relative mRNA expression level of the apoptin compared to endogenous GADPH.

### Hoechst 33324 staining

HepG2, PLC, Huh-7, WI38 and L-02 cells were seeded in 6-well culture plates and infected with AD55-Apoptin, AD55 and ONYX-015 at a MOI of 5, uninfected cells served as control. After 48 hours, cells were treated with the apoptosis-Hoechst 33324 staining kit (Beyotime) for 5-10 min as described protocol, washed with PBS twice, and observed under a fluorescence microscope.

### Flow cytometric analysis

Cells infected with various adenoviruses were trypsinized and washed once with complete medium. Aliquots of cells (5 × 10^5^) were resuspended in 500 ml of binding buffer and stained with fluorescein isothiocyanate (FITC)-labeled annexin V (BioVision, Palo Alto, CA) according to the manufacturer's instructions. A fluorescence-activated cell-sorting (FACS; BD Biosciences, San Jose, CA) assay was performed immediately after staining.

### Studies on xenograft tumors in nude mice

All animals used in these experiments were maintained in the institutional facilities in accordance with regulations and standards of the US Department of Agriculture and the National Institutes of Health. Female BALB/c nude mice at 4-5 weeks obtained from the Animal Research Committee of the Institute of Biochemistry and Cell Biology (Shanghai, China) were used in all of the experiments. Huh-7 cells (1 × 10^7^) were injected subcutaneously into the lower right flank of female nude mice. After about two weeks, tumor xenografts model was established. Each group was at least comprised of eight animals and the tumor growth was monitored and measured with a vernier caliper and tumor volume (V) was calculated using the formula V (mm^3^) = 1/2 × length(mm)×width(mm)^2^. When the tumors were about 425 mm^3 ^in size, mice were randomized into four groups and a daily dose of 3 × 10^8 ^plaque-forming unit (PFU) of examined viruses AD55-Apoptin, AD55 and ONYX-015 suspended in 100 μl of PBS or 100 μl PBS alone was administrated intratumorally once everyday for a total of five. The tumors were harvested at the fourth day post treatment with adenovirus for H&E staining, Immunohistochemical study and The TdT-mediated dUTP-biotin nick end-labeling (TUNEL) staining.

### Immunohistochemical (IHC) study

For IHC analysis, tumors on day 4 post-treatment were harvested and fixed in 4% paraformaldehyde, embedded in paraffin and cut in 4 um sections. These sections were stained with goat monoclonal anti-adenoviral hexon antibody at a 1:200 dilution, respectively. The slides were then washed with PBS and incubated with the avidin-biotin-peroxidase complex reagent (Vector Laboratories, Burlingame, CA) and detected with diaminobenzidine tetrahydrochloride [27] solution containing 0.006% hydrogen peroxide. Hematoxylin was used as a counterstain. Tissue sections stained without primary antibodies were used as negative controls.

### TUNEL apoptosis assay

TUNEL assay was used for detection of apoptotic cells. For this purpose, the *in situ *cell apoptosis detection kit (Roche, USA) was used. The staining was carried out according to the manufacturer's procedures. Tissue sections in PBS group were stained and served as positive controls. The TUNEL reaction preferentially labels DNA strand breaks generated during apoptosis, and allows discrimination of apoptosis from necrosis and primary DNA strand breaks induced by apoptotic agents.

### ALT and AST assay after targeting gene-virus treatment in nude mice

As for the each treated group(n = 3), after the viruses were intratumorally injected into nude mice a month later, the nude mice were killed and collected their blood through eyeball and liver function was examined according to the related standard procedures [28].

### Statistical analysis

The statistical significance of experimental results was calculated by analysis of variance (ANOVA) and student t-test. Datas were considered statistically significant at *P *< 0.05.

## Results

### Construction and characterization of the recombinant AD55-Apoptin

**Figure 1:**
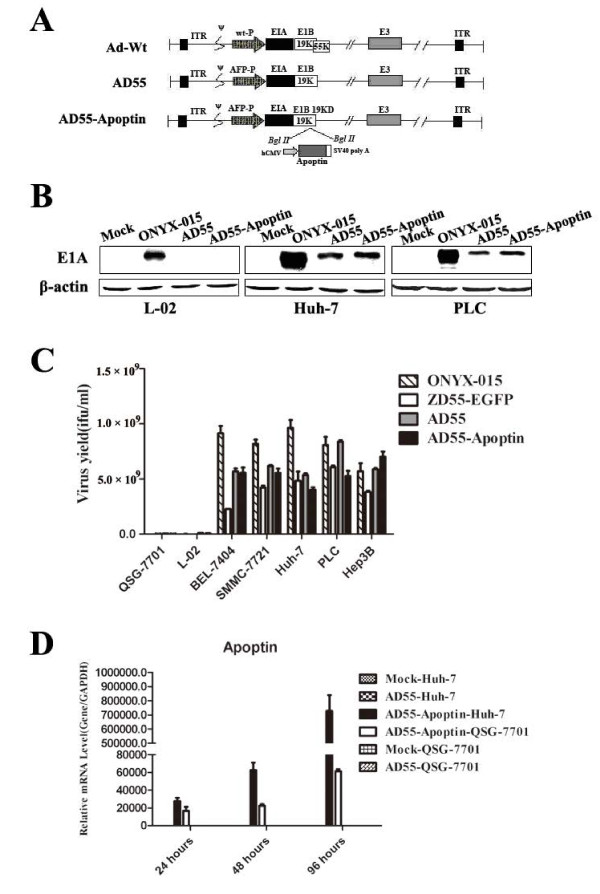
**Construction of the dual regulated oncolytic adenovirus AD55-Apoptin and its selective replication in tumor cells**. **A**. Schematic structure of recombinant adenoviruses. Compared with E1B 55KD-deficient adenovirus ZD55, the dual-regulated adenovirus AD55-Apoptin had been further modified with both the E1A promoter replaced by eAFP and the E1b55KD deletion. Then, the Apoptin expression cassette controlled by human cytomegalovirus (HCMV) promoter was obversely inserted to form AD55-Apoptin. ITR is the inverted terminal repeat sequence. **B**. Detection of E1A levels of recombinant oncolytic adenoviruses. L-02, Huh-7 and PLC was infected with ONYX-015, AD55 and AD55-Apoptin at the MOI of 5, after 48 hours, Western Blotting was conducted to detect E1A protein expression, β-actin was used as a protein loading control. **C**. 3.5 × 10^5 ^cells were plated into six-well plates. After 24 h, the cells were infected with 10 MOIs of AD55-Apoptin or AD55 or ONYX-015 or ZD55-EGFP, respectively. After an additional 48 h, medium and cells were scraped into 1.5 ml Eppendorf tube and subjected to three-thaw cycles. The collected supernatant was tested for virus production by standard TCID50 assay on 293 cells. Progeny viruses from 1 MOI of virus were calculated. The results were the average of two independent experiments. **D**. Huh-7 and QSG-7701 cells were infected with AD55 or AD55-Apoptin at the MOI of 10 for 24, 48 and 96 hours, respectively. The total RNA was collected at the indicated time and reverse transcripted into cDNA, then a real time quantitative PCR was done with the Apoptin or GAPDH primers.

The dual regulated oncolytic adenovirus AD55 based on oncolytic adenovirus ZD55 with E1B 55KD deletion was constructed by using HCC-specific eAFP promoter to control E1A gene. Then, the antitumor gene Apoptin was inserted into E1B region and formed AD55-Apoptin. The construction of AD55 and AD55-Apoptin were shown in Figure 1A.

To evaluate the characterization of the constructs, we detected the tumor-specific expression of adenovirus E1A by Western blot assay. The normal hepatocelluar line L-02 and two HCC cell lines Huh-7 and PLC were infected with ONYX-015, AD55and AD55-Apoptin at a MOI of 5 for 48 hours, then the E1A protein expression was detected. The results showed that the dual regulated oncolytic adenovirus AD55 and AD55-Apoptin only expressed E1A protein in Huh-7 and PLC HCC cells but not in L-02 normal cells compared to that of ONYX-015 with positive expression level, the mock group was as a negative control (Figure 1B). It demonstrated that this dual regulated constructs can specifically replicates in HCC cells.

To examine whether the transgene and modified genome of adenovirus could interfere with the selective replicative ability of recombinant adenoviruses in different cell lines, a progeny assay was performed in tumor cells (BEL7404, PLC, Hep3B, SMMC-7721 and Huh-7) and normal liver cell lines (L-02, QSG-7701) infected with different constructs including (ONYX-015, ZD55-EGFP, AD55 and AD55-Apoptin). As shown in Figure 1C, the above viruses can easily replicate in infected liver cancer cells with more virus progeny yield compared to the normal liver cells with reduced replicative capacity. These data indicated that insertion of *apoptin *gene and deletion of E1B did not strongly affect the selective replicative ability of AD55-Apoptin in cancer cell compared with the vector AD55, although its replicative capacity was different from the other constrcuts.

Moreover, in order to examine the expression of *apoptin *gene, real time PCR assay was done after infection of Huh-7 or QSG-7701 cells with AD55-Apoptin or AD55 at a MOI of 10 for the indicated hours in Figure 1D. The results indicated that the expression of apoptin was remarkable time-dependent in Huh-7 cells but not in normal liver QSG-7701 cells, these data further supported AD55-Apoptin can selectively target the HCC cells.

### Cytotoxicity of AD55-Apoptin in tumor cells but not in normal cells

**Figure 2:**
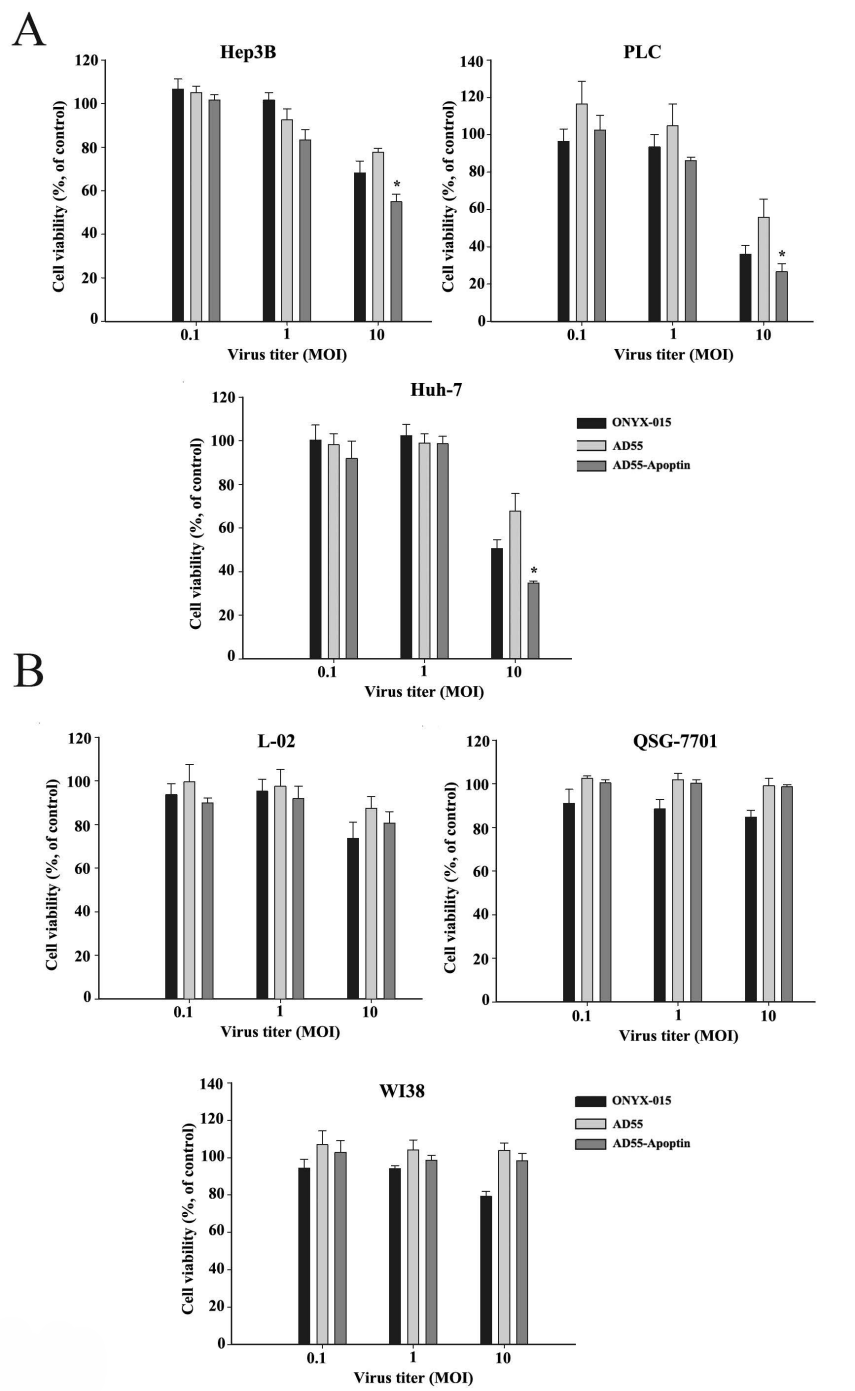
**MTT assay detected cell viability of tumor cells and normal cells infected with increasing MOIs of recombinant oncolytic adenovirus**. A.Tumor cells were infected with ONYX-015, AD55 and AD55-Apoptin at the increasing MOIs (0.1, 1, 10), after 4 days, cell viability was determined by MTT assay. B. Cell viability of normal cells (L-02, WI38, QSG-7701) infected with the above three different viruses, Datas were shown as means ± SD (error bars) of triplicate experiments,* p < 0.05, compared with the AD55 treatment group.

To validate the cytotoxicity of AD55-Apoptin, the HCC cell lines (Hep3B, PLC and Huh-7) and normal cell lines (L-02, QSG-7701 and WI38) were infected with indicated adenovirus at MOI of 0.1, 1, 10 respectively. 4 days later, cytotoxicity was determined by MTT assay. As shown in Figure 2A, AD55-Apoptin, AD55 and ONYX-015 all could induce dose-dependent cytotoxicity in these tumor cell lines, Nevertheless AD55-Apoptin exhibited higher cytotoxicity (p < 0.05) than that of AD55 and ONYX-015, but showed no obvious cytotoxicity to the normal cell lines compared to ONYX-015 and AD55 (Figure 2B), although the antitumor effect of the vector AD55 was not strong than that of ONYX-015. The results suggested that AD55-Apoptin had an obvious cytotoxic effect on different hepatocarcinoma cells *in vitro *but had no or little influence on normal cells.

**Figure 3:**
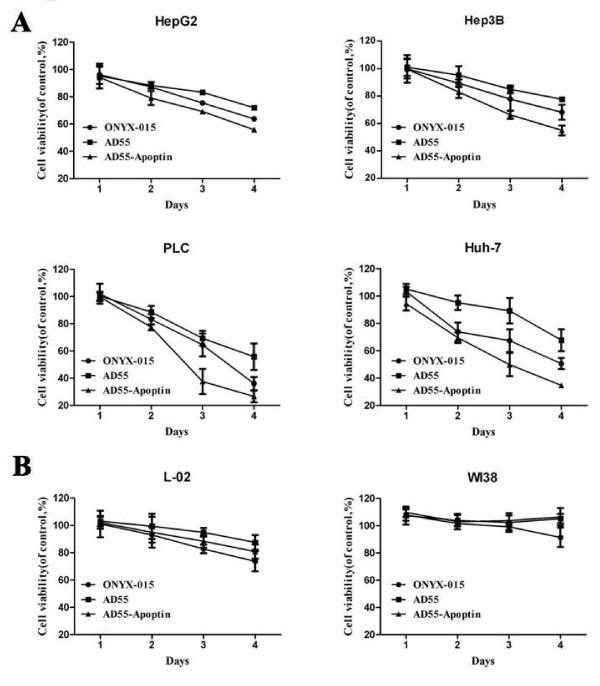
**AD55-Apoptin showed the selective anti-hepatoma effect in a time-dependent manner**. A. Tumor cells (Huh7, PLC, Hep3B, HepG2) were infected with ONYX-015, AD55 and AD55-Apoptin at the MOI of 10 for 1,2,3,4 days, respectively. At the indicated time, cell viability was determined by MTT assay. B. According to the above similar method, the cell viability of normal cells infected with various viruses was detected by MTT assay. Datas are presented as means ± SD (error bars) of triplicate experiments.

Additionally, the cell-killing effect was also observed by MTT assay in the indicated time course (Figure 3). HCC cell lines HepG2, Hep3B, PLC, Huh-7 and normal cell lines L-02 and WI38 were infected with ONYX-015, AD55 and AD55-Apoptin at the MOI of 10, respectively. And the antitumor ability and safety were evaluated at day1, 2, 3 and 4. As shown in Figure 3, AD55-Apoptin displayed much higher toxicity than that of ONYX-015 and AD55 in HCC cells but no notable toxicity in normal cells WI38, although a little bit higher toxicity than that of AD55 in L-02 cells.

### Mechanism of apoptosis induced by AD55-Apoptin in Huh-7 cells

**Figure 4:**
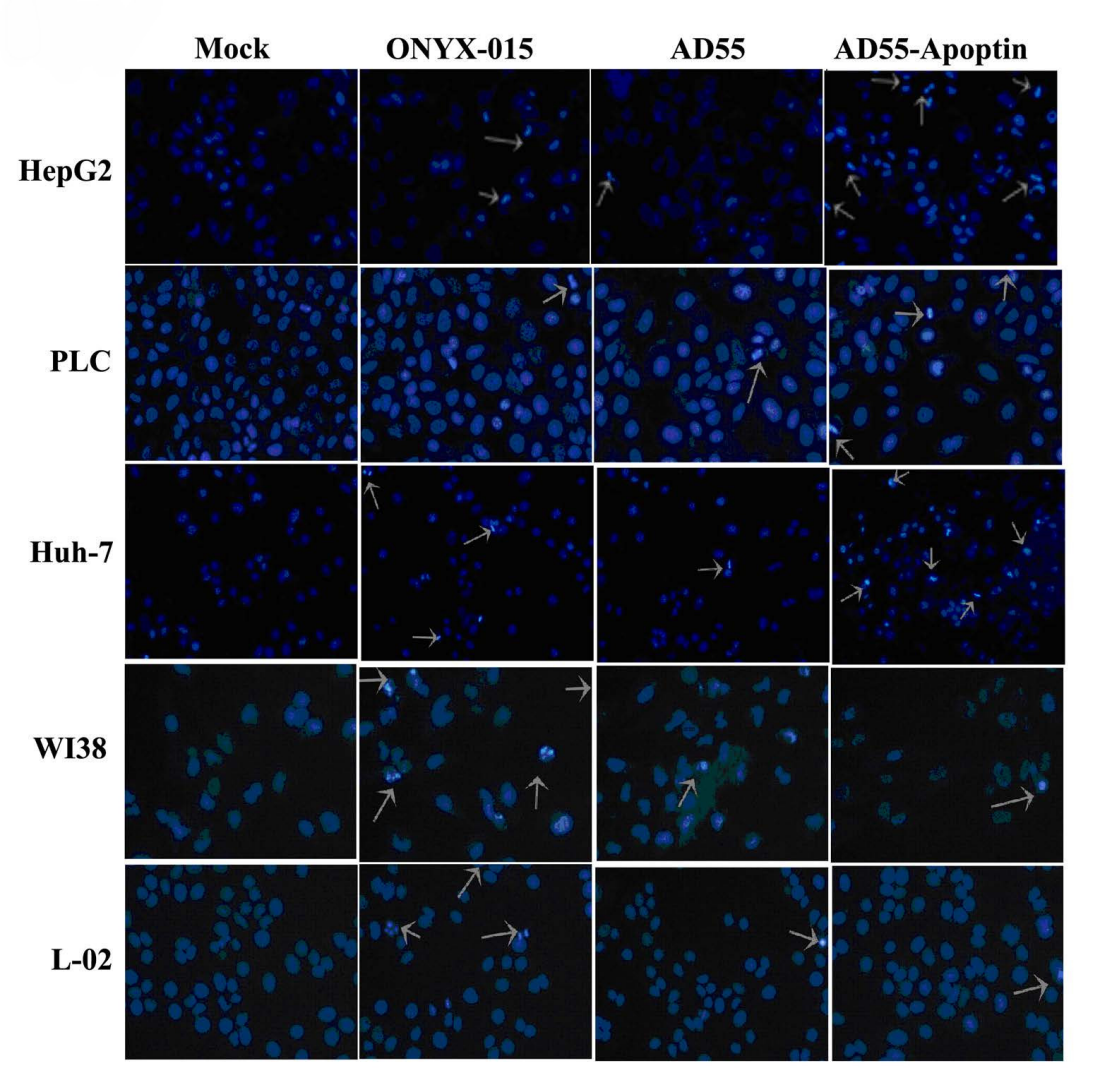
**Hoechst 33342 stanning for the apoptotic cells infected with the various viruses**. Tumor cells or normal cells were infected with AD55, AD55-Apoptin and ONYX-015 at the MOI of 5, respectively. After 48 hours, cells were incubated with Hoechst 33342 for 30 min and observed under a fluorescence microscope (arrows indicated apoptotic cells). (Original magnification: ×200).

To further investigate whether AD55-Apoptin killed the tumor cells through apoptosis, HCC cells (HepG2, PLC and Huh-7) and normal cells (WI38 and L-02) were analyzed with apoptotic cells stained by Hoechst 33342 after treatment with AD55-Apoptin, AD55 and ONYX-015 at a MOI of 5 for 48 h. The results clearly addressed that AD55-Apoptin induced more remarkable apoptotic morphological changes such as chromatin condensation, formation of apoptotic body than that of AD55 and ONYX-015 in Huh-7 cells but much less in normal cells L-02 and WI38 in Figure 4. Moreover, the other HCC cells such as PLC, HepG2 also showed the apoptic cells treated with AD55-Apoptin, although it did not seem obvious.

**Figure 5:**
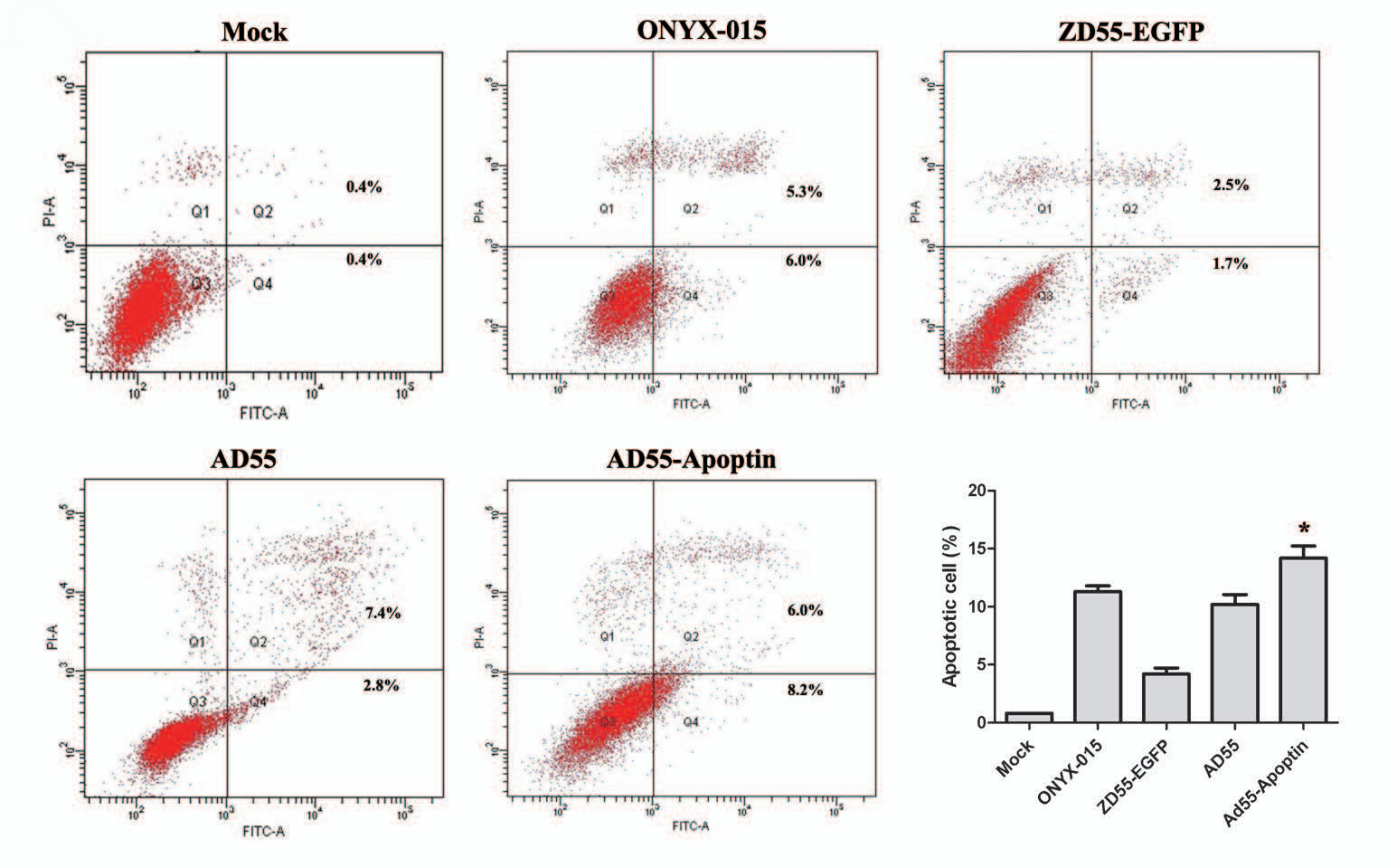
**Mechanism of apoptosis is mediated by AD55-Apoptin in Huh-7 cells**. Huh-7 cells infected with ZD55-EGFP or ONYX-015 or AD55 or AD55-Apoptin at the MOI of 10, respectively. After 48 hours, the cells were trypsinized and washed once with complete medium. Aliquots of cells (5 × 10^5^) were resuspended in 500 ml of binding buffer and stained with fluorescein isothiocyanate (FITC)-labeled annexin V kit, according to the manufacturer's instructions. A fluorescence-activated cell-sorting assay was performed immediately after staining. Mean ± SD;*.p < 0.01, compared with AD55 treated group.

In addition, fluorescence activated cell sorting (FACS) assay showed AD55-Apoptin can strongly induce the apoptosis of Huh-7 cell compared to the other groups in Figure 5. These data further confirmed the mechanism of apoptosis can account for the anti-hepatoma effects of AD55-Apoptin.

### Antitumor efficacy of AD55-Apoptin in nude mice

**Figure 6:**
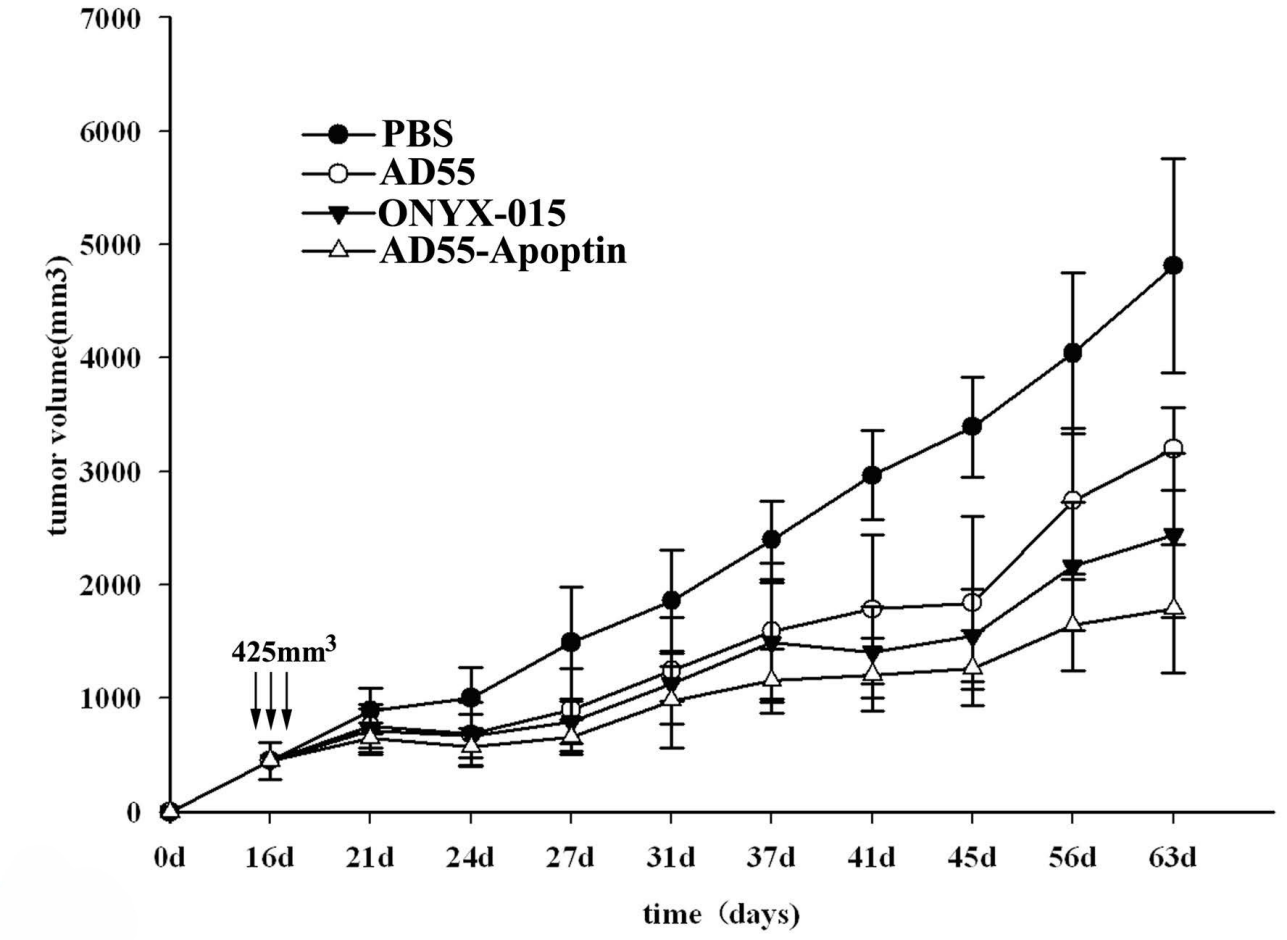
**Antitumor effect of AD55-Apoptin in nude mice xenograft model**. Tumors were established by injecting Huh-7 cells subcutaneously into the right flank of nude mice. When tumors reached about 425 mm^3^, the mice were divided into four groups (n = 8) and treated with five consecutive daily intratumoral injections of PBS or ONYX-015, AD55 and AD55-Apoptin at 3 × 10^8 ^PFU/dose everyday per nude mice The 16^th ^day was the starting time of treatment. Datas were showed as means ± SD.

To determine the antitumor activity of AD55-Apoptin *in vivo*, a model of Huh-7 human liver tumor xenograft was established in nude mice. When the tumors volume reached to about 425 mm^3 ^which was more closed to advanced HCC, PBS, AD55-Apoptin, AD55 and ONYX-015 (3 × 10^8 ^PFU/dose) in 100 μl were injected intratumorally everyday by five times totally. Mice treated with AD55-Apoptin exhibited statistically significant suppression of hepatocarcinoma development compared to ONYX-015 (P < 0.05) or AD55 (P < 0.05) or PBS treated mice ((P < 0.01)) in Figure 6, although the antitumor effect of ONYX-015 was superior to that of AD55, which was consistent with the related MTT results *in vitro*.

### Evidence for AD55-Apoptin on tumor growth inhibition in vivo

**Figure 7:**
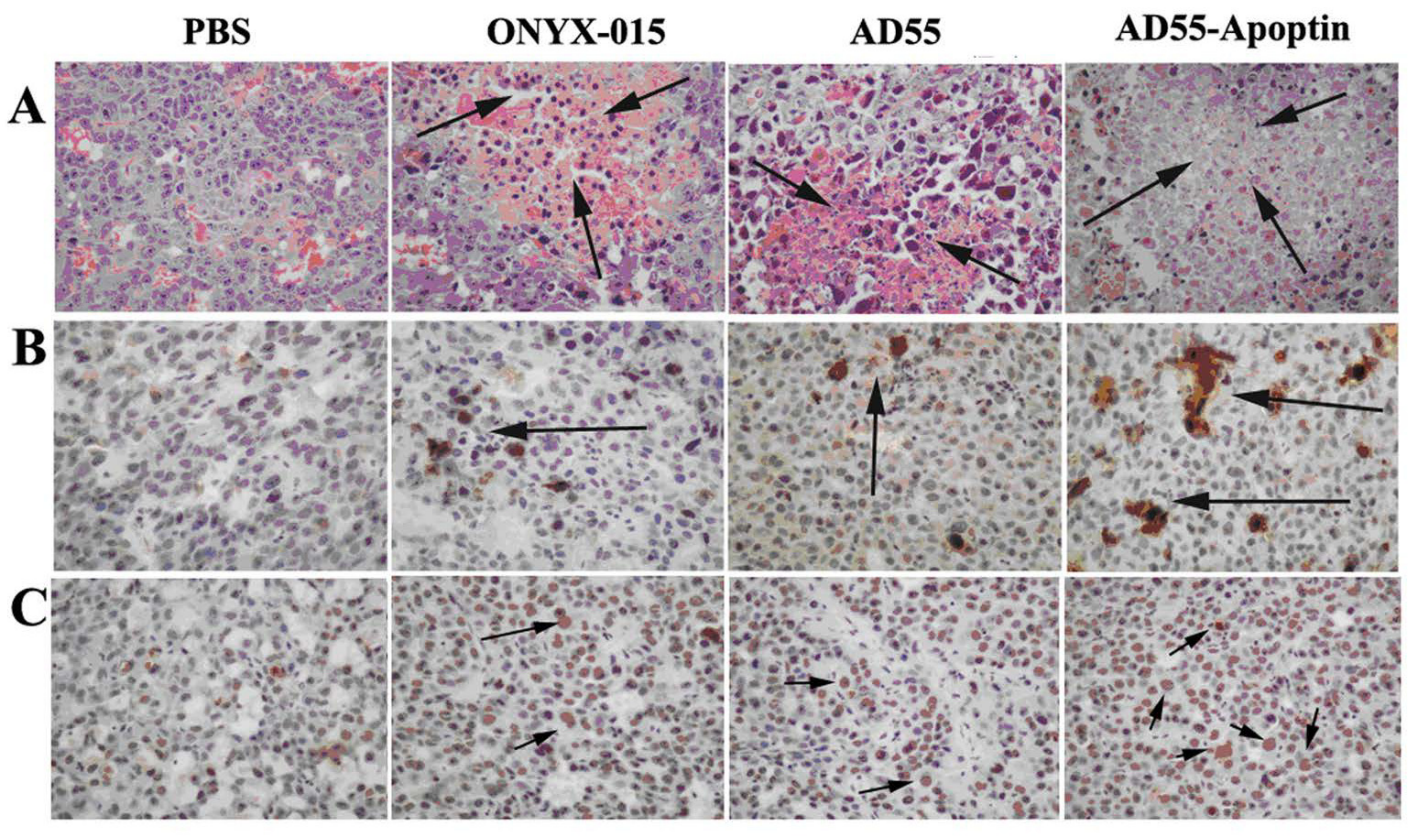
**Evidences of antitumor efficacy of AD55-Apoptin in vivo**. The pathologic detection by H.E, IHC and TUNEL staining assay were performed. Subcutaneous Huh-7 tumors receiving various treatments were harvested on the 4th day after infection with viruses. Tumor sections were treated as described above methods and materials. A. H.E staining assay. partial area of necrosis have been detected in the sections treated with ONYX-015, AD55, AD55-Apoptin (indicated with the black arrows), compared to the PBS group(×100, original magnification). B. The adenovirus hexon protein as an indicator of adenovirus in vivo was detected by IHC analysis as described above methods and materials. (×400, original magnification). Hexon expression was positive (arrows indicated) in three groups treated with ONYX-015 or AD55 or AD55-Apoptin, respectively. C. The terminal deoxynucleotidyl transferase-mediated dUTP nick end labeling (TUNEL) assay for detection of apoptotic cells in tumor sections treated with PBS or the above various recombinant viruses (×400, original magnification). The black arrows indicated the obvious apoptotic cells in the AD55-Apoptin treated group than that of AD55, ONYX-015 or PBS treated group.

Hematoxylin and eosin (H&E) staining showed that injections of AD55-Apoptin caused profound cell death and necrosis symptom in tumor mass than that of ONYX-015 or AD55, the PBS-treated group as a negative control (Figure 7A). The results of immunohistochemical analysis exhibited strong hexon expression in tumor cells treated with AD55-Apoptin but less positive in those treated with ONYX-015 or AD55, the PBS-treated group was as negative control (Figure 7B). Additionally, terminal deoxynucleotidyl transferase-mediated dUTP nick end labeling (TUNEL) staining further confirmed AD55-Apoptin induced more obvious apoptotic cells (arrows indicated the brown stained cells)sis in tumors whereas the apoptotic cells in the group treated with ONYX-015 or AD55 were less (Figure 7C).

Moreover, the mice liver function was examined one month later after injection of PBS or ONYX-015 or AD55-Apoptin respectively. The results demonstrated that AD55-Apoptin had less impair effect on the liver function by AST/ALT at 1.22 than ONYX-015 at 3.00(p < 0.01) or even less than on treatment PBS group at 2.80(p < 0.01) (the detailed data not show). It further demonstrated the safety of AD55-Apoptin on hepatocarcinoma therapy.

## Discussion

The conditionally replicating oncolytic adenovirus is exploited as a powerful agent for cancer therapy. The most famous and researched is ONYX-015 which already entered phase III clinical trial. Originally, ONYX-15 was convinced selective replication in p53-deficient tumor cells by thinking of viral E1b-55 kDa protein switch off p53 [29]. But later more exceptions or even contraries were found, such as ONYX-15 replicated in U87 cells(p53^+^) [30] as same as wild type did. Although recently it was clarified that late viral RNA export, rather than p53 inactivation, determines ONYX-015 tumor selectivity [7], what's more, a novel pathway showed that the adenoviral protein, E4-ORF3, induced histone methylation silences p53 targets [31]. But the precise mechanism remains unknown. Furthermore ONYX-015 still can replicate in primary hepatocytes [7,32], consistent with our studies shown ONYX-015 can kill normal hepatocellular cells L-02 to some extent (Figures 3 and 4).

Hence, in our study we replaced the native E1A promoter with HCC specific promoter AFP promoter based on ZD55 to make it more specifically replicate in hepatocarcinoma cells. Our data showed that AD55 induced much less damage on normal cells although at the expense of some killing effect on tumor cells(Figure 3). Even though it is known that cellular promoters do not keep the proper fidelity in the viral genome. Low levels of E1a protein may be sufficient for replication, thus preventing specificity [33,34]. Therefore, in order to augment the antitumor effect but also keep the specificity to the fullest extent of AD55, it is worthwhile to explore a gene which possesses not only remarkable antitumor features but also high tumor selecting. Apoptin as a small apoptosis-inducing protein derived from chicken anemia virus (CAV) has such properties.

Piles of reports have shown Apoptin induced the selective death in various of human tumor cells including melanoma, hepatoma, lymphoma, cholangiocarcinoma, colon carcinoma, breast and lung cancer and other types [35,36], even also in SV40-transformed fibroblasts or UV-irradiated cells from individuals with hereditary cancer-prone syndromes [13,37], but not in normal cells, non-transformed cells such as human endothelial cells, hepatocytes, hematopoietic stem cells, keratinocytes, or smooth muscle cells [38]. Furthermore the efficiency and safety of Apoptin on tumor therapy were further testified *in vivo *by mice models [21,39]. Previous studies have well established Apoptin induces cell death though the intrinsic mitochondrial pathway. Apoptin can trigger the release of cytochrome c which at last induces caspase 9 activation via combination with Apaf-1 [16,40]. While the mechanism by which Apoptin is able to distinguish between tumor and normal cells remains unclear, but seems to correlate with its cellular localization. Recently, Maddika et al indicated PI3K/Akt/CDK2 pathway plays a pivotal role in Apoptin-induced apoptosis. Their results revealed that Apoptin interacts with PI3K/Akt results in its nuclear translocation and sustained activation which initiates apoptosis instead of a survival response, on the other hand, the activated Akt further activates CDK2 which directly phosphorylates apoptin to lead to the accumulation of apoptin in nuclear, this is crucially required for Apoptin-induced cell death. The fact suggested that inhibitors of PI3-kinase or Akt inhibited CDK2 activation hence to protect cells from Apoptin-induce cell death [41,42]. Considering the abnormal PI3K/Akt activation in various type of cancer cells [43], this at least can partially elucidate the specificity and efficiency of apoptin on killing tumor cells. Nevertheless, more details still need to be explored.

Additionally, some other properties of apoptin make it more attractive for tumor gene therapy, including the ability to induce tumor-specific apoptosis independently of p53 [14] which is deficient in many cancers that are resistant to chemotherapeutic agent. Another is that in certain tumor cell lines it mediated cell death that is independent of the Bcl-2 status and even stimulated by Bcl-2 [16], although the role of Bcl-2 in Apoptin-induced apoptosis is still a matter of debate. The apparent controversy as to the role of Bcl-2 proteins may arise from the differential involvement of Nur77, in Apoptin-induced cell death. It has been shown that Nur77 can bind to Bcl-2 and change its properties from an anti-apoptotic to a pro-apoptotic molecule, leading to the activation of mitochondrial death pathway [44]. Because Nur77 expresses in different cell types at very varying levels, this might explain the opposite effects of Bcl-2 on Apoptin-induced cell death. Besides, Maddika et al indeed proposed a role of Nur77 in Apoptin mediated apoptosis [45]. Based on above all, Apoptin is bona fide a promising candidate for our trial.

As shown in our study, AD55-Apoptin demonstrated a much stronger capability to induce apoptosis of hepatocarcinoma cells than that of ONYX-015 and AD55, however, it had no or little impair on normal cells (Figures 2, 3, 4A), no matter in a dose dependent manner (Figure 2) or time dependent manner (Figure 3). Moreover, the efficiency and safety of AD55-Apoptin were demonstrated in HCC cell line Huh-7 xenograft models (Figure 6). It can even inhibit the hepatoma tumor growth with a large volume up to about 425 mm^3 ^in nude mice xenograft model because a proper beginning tumor volume in a routine xenograft model at volume of 80-120 mm^3 ^or a litter bigger of 100-150 mm^3 ^was sensitive and effective to the recombinant adenovirus. Pathological examination further confirmed the replicative progeny virions of AD55-Apoptin expressed the adenovirus hexon protein specifically in xenograft cancer cells and induced more apoptosis than AD55 and ONYX-015(Figure 7). In view of connection between Apoptin and PI3K/Akt, some antitumor chemotherapeutic agents such as methotrexate or docetaxel [46] or paclitaxel [47] which sensitize tumor cells for apoptosis under prolonged nuclear Akt/CDK2 activation may can be used together with AD55-Apoptin to get a even better effect.

## Conclusion

AD55-Apoptin was successfully generated and more powerful and safe on hepatocarcinoma therapy than AD55 or even classical ONYX-015. It throws some light on the strategy of Cancer Targeting Gene-Viro-Therapy and may provide some referenced value for clinic cancer therapeutic research.

## Competing interests

The authors declare that they have no competing interests.

## Authors' contributions

KJZ, JQ, SBW and YY performed experiments. KJZ and YY designed the study and wrote the manuscript. All authors read and approved the final manuscript.
